# Plasma erythritol and cardiovascular risk: is there evidence for an association with dietary intake?

**DOI:** 10.3389/fnut.2023.1195521

**Published:** 2023-05-23

**Authors:** Thorsten Cramer, Ulrike Gonder, Barbara Kofler

**Affiliations:** ^1^Department of General, Visceral and Transplantation Surgery, RWTH University Hospital, Aachen, Germany; ^2^Nutritionist, Freelance Science Writer, Hünstetten, Germany; ^3^Research Program for Receptor Biochemistry and Tumor Metabolism, Department of Pediatrics, University Hospital of the Paracelsus Medical University, Salzburg, Austria

**Keywords:** erythritol, food supplement, cardiovascular risk, epidemiology, platelet function, pentose phoshate pathway

## Introduction

The sugar alcohol erythritol is naturally occurring in some fruits such as grapes, melons, pears and fermented products like soy sauce, red wine and cheese (1). In addition, erythritol is an endogenously occurring polyol (2). A study published in early 2023 in *Nature Medicine* entitled “The artificial sweetener erythritol and cardiovascular event risk” (3) has caused quite some controversy and uncertainty in the nutrition field *inter alia* clinicians and dieticians as well as the general public. The data presented in this paper seem to be interpreted in a way that consumption of erythritol leads to a significantly increased risk of suffering “cardiovascular events” [death or non-fatal heart attack or non-fatal stroke, summarized as “major adverse cardiovascular events” (MACE)].

Here, we first provide a summary of the results of the study by Witkowski and Nemet et al. (3) and a critical evaluation of whether or not there are enough research data available to conclude with confidence, whether or not dietary erythritol intake is associated with enhanced cardiovascular risk or death. In addition, we briefly discuss endogenous erythritol synthesis and selected studies reporting the effect of erythritol intake on other diseases.

## Plasma erythritol levels and cardiovascular events in patient cohorts

First, the authors used blood samples from a large cohort study (>1,100 subjects with high prevalence of cardiovascular disease or risk factors) to perform an “untargeted metabolomics” analysis using a state of the art, mass spectrometry-based analysis to measure (= identify and quantify) a very large number of features in the blood of the subjects. As such, several features (peak or signal that represents a chemical compound) from the group of polyols (organic compounds containing several hydroxy groups (-OH), also known as polyalcohols) were detected. In view of the fact that many sugar substitutes fall into the group of polyols (e.g., sorbitol, xylitol, and erythritol), the authors focused their further investigation on erythritol. Comparison of the measured levels of erythritol with the clinical courses from the cohort patients showed that subjects who experienced MACE within 3 years of follow-up had statistically significantly higher erythritol concentrations in their blood [see Figure 1 in (3)].

In the next step of their investigation, the authors established a “targeted metabolomics” analysis in order to achieve a more precise quantification of erythritol. This was necessary because the above mentioned “untargeted metabolomics” analysis did not allow an absolutely reliable differentiation between erythritol and structurally related polyols. The improved methodology was used to analyse blood samples from two further cohort studies from the US and Europe (>2,200 and >800 subjects), again with high prevalence of cardiovascular disease and risk factors, respectively. Here, a similar picture emerged, i.e., subjects at increased risk of MACE had statistically significantly higher blood erythritol concentrations [see Figures 1 and 2 in (3)].

It is interesting to note that the concentration of erythritol in the plasma of the subjects increased with age in all three cohorts. The subjects with the highest erythritol levels (top quartile) were on average 10–14 years older and had a higher prevalence of diabetes than the subjects with the lowest values [lowest quartile, see Tables S1–3 in (3)]. Still, after stratification of the results for confounding factors such as age, body mass index and other risk factors, higher erythritol concentration remained as an independent risk factor in all cohorts when men and women were analyzed together. However, when sexes were analyzed separately, no significant association was observed in two of the three cohorts in women [Tables S7 and S8 in (3)].

## Effect of erythritol on platelet function *in vivo* and *in vitro*

Based on the epidemiological findings, the authors hypothesized that erythritol may modify the function of blood platelets. The background to this hypothesis is the well-established causal role of increased aggregation of platelets for the formation of blood clots (thrombi) in blood vessels supplying the heart and the brain, respectively, in the pathophysiology of myocardial infarction and stroke. The authors used two different preparations of platelets (platelet-rich plasma and washed human platelets) from healthy volunteers for *in vitro* analyses. Erythritol significantly increased the aggregation of human platelets in a dose-dependent manner [see Figure 3a in (3)]. Furthermore, erythritol increased thrombus formation in a model examining human blood under conditions of physiological shear forces. Finally, these results were further verified in a mouse model. Again, significant stimulatory effects of erythritol on blood clot formation were found [see Figure 4b in (3)].

## Pharmacokinetics of exogenous erythritol in human plasma

Lastly, the authors conducted a pharmacokinetic study with 8 healthy volunteers. In this study, volunteers had to drink 300 ml of a beverage sweetened with 30 g of erythritol within 2 min following an overnight fast. Subsequently, blood samples were taken at specific time points and the concentration of erythritol was determined. Within the first 30 min, there was an up to 1,000-fold increase in erythritol concentration (basal values: 3.84 (3.27–4.14) μM) vs. 5.85 (4.30–7.68) mM [median (25th and 75th percentiles) at 30 min]. Remarkably, significantly elevated erythritol concentrations were detectable in the blood of these volunteers up to 48 h after consumption of the sweetened beverage (3).

## Discussion

Cohort studies are an excellent tool for generating hypotheses. However, causal relationships cannot be proven by cohort studies (4). The authors were totally aware of this limitation and they also mention it in the discussion (“... these studies can only show association and not causation”). Therefore, this study cannot clarify whether the consumption of erythritol leads to an increased risk of heart attack and stroke, and because no erythritol consumption data were available no definitive recommendation to reduce the consumption of erythritol can be derived from this study.

The subjects from the cohort studies had shown increased cardiovascular risk [see supplementary tables in (3)]. Therefore, the results of the article by Witkowski and Nemet et al. do not allow an assessment of the effects of erythritol on the development of heart attacks or strokes for healthy people who consume erythritol occasionally, or even regularly, e.g. as part of a carbohydrate-reduced diet. The authors of the study are also aware of these correlations, as they emphasize several times that their results only apply to people with increased cardiovascular risk.

Erythritol found in human blood can have two origins: Either it is ingested with food (= exogenous) or it originates from the metabolism of the subject (= endogenous). Erythritol is constitutively synthesized in humans from glucose via the so-called pentose-phosphate pathway, a side arm of glycolysis (2). As discussed in depth in a recent review by Mazi *et al*., elevated erythritol levels might be an indicator of pentose phosphate pathway dysregulation resulting from glucose and fructose rich diets (5). Interestingly, endogenous erythritol synthesis is also elevated in response to oxidative stress (6). It is known that the pentose-phosphate pathway is more active in subjects with pre-existing cardiovascular disease (1, 7). This could therefore explain the elevated erythritol concentrations measured in the cohorts, without consumption via food being a factor. This is also supported by the fact that blood samples were obtained at a period of time when erythritol consumption was much rarer than, say, today and was completely absent in the first cohort (2007–2009). Two other studies including samples from 1987 to 1989 were conducted well before erythritol was available as a sweetener, reporting elevated levels of it with incident diabetes and coronary artery disease (8, 9).

A limitation of the study is the fact it was not possible to determine whether the erythritol levels in plasma were influenced by exogenous consumption, since no information on the diet or composition of the food in the days prior to blood samples being drawn, was collected from the subjects. This is a noteworthy caveat. In addition, it should be noted that the older individuals with higher erythritol levels also had greater pre-existing risk factors including a higher prevalence of diabetes (3). It is also unknown whether endogenous erythritol production generally increases with age.

We consider the “Erythritol intervention study” conducted by the authors to be another weakness of the article. As described in detail above, the “intervention” consists of the consumption of a 300 ml drink sweetened with 30 g of erythritol within 2 min on an empty stomach. This is a typical “study situation” and rather rarely corresponds to a representative breakfast meal in real life. Erythritol-containing beverages and foods are typically consumed throughout the day, and not within a very short timeframe. According to the commission regulation [(EU) 2015/1832 of 12 October 2015 amending Annex II to Regulation (EC) No 1333/2008] erythritol is allowed as a flavor enhancer in energy-reduced or with no added sugars flavored drinks at a maximum level of 1.6 %. Thus, 300 ml of a drink can contain a maximum of 4.8 g of erythritol. Moreover, erythritol is very often present within a food matrix that has a delaying effect on postprandial absorption. It is therefore questionable whether the reported plasma concentrations also occur under “real life” conditions. Furthermore, it is completely unclear to what extent different diets *inter alia* a low-carb or ketogenic diet affect the postprandial absorption of erythritol. The intestinal microbiome is critically involved in the digestion and absorption of various substances, and carbohydrate-reduced diets may critically alter the composition of the intestinal microbiome. In contrast to other sugar alcohols erythritol is not fermented by the gut microbiome (10).

A long-term rat study with a daily erythritol dose up to 5.2 g/kg for 2 years, which is even 100-fold more than the amount used in the mouse thrombosis model (25 mg/kg), did not affect kidney function, cancer incidence and survival of the animals (11). Notwithstanding, long-term clinical studies with erythritol in humans are lacking, a pilot study tested a daily intake of 36 g/day for 4 weeks in 24 type 2 diabetics, which led to reduced arterial stiffness and improved endothelial function (12). As the dose provided by the long term study is 100-fold higher than the dose used in the *in vivo* study of Witkowski and Nemet *et al*. (3) any adverse effects of erythritol on platelet function might be outweighed by potential beneficial effects (5), or at the very least may not influence longevity.

In summary, the authors must be credited for not overstating the results of their study, but explicitly pointing out in the discussion [especially in the last paragraph, page 7 in (3)] that their results suggest that clinical trials are needed to investigate the relevance of erythritol (and other sugar substitutes) for the development of cardiovascular disease using sufficient duration and relevant clinical end points. Such a differentiated and transparent presentation is remarkable and (unfortunately) rather not the rule in nutritional studies.

As so often is the case with nutritional studies, the results do not permit the derivation of recommendations. As explained above, no causal relationship between erythritol consumption and cardiovascular risk can be derived from the epidemiological study results (3, 8, 9). Limiting the consumption of erythritol remains an individual decision. We are aware that this is an unsatisfactory statement, but without results from well-designed and transparently analyzed clinical trials, a definitive recommendation is, for the time being, not possible.

## Author contributions

TC and BK contributed to the conception and design of the opinion-study and drafted the manuscript first. UG provided additional input and edited the manuscript. All authors contributed to the article and approved the submitted version.
